# Prevalence of symptoms in glioma patients throughout the disease trajectory: a systematic review

**DOI:** 10.1007/s11060-018-03015-9

**Published:** 2018-10-30

**Authors:** Margriet IJzerman-Korevaar, Tom J. Snijders, Alexander de Graeff, Saskia C. C. M. Teunissen, Filip Y. F. de Vos

**Affiliations:** 1 Department of Medical Oncology, Cancer Center, University Medical Center Utrecht, University Utrecht, Heidelberglaan 100, 3584 CX Utrecht, The Netherlands; 2 Department of Neurology, Brain Center Rudolf Magnus, University Medical Center Utrecht, University Utrecht, Heidelberglaan 100, 3584 CX Utrecht, The Netherlands; 3 Julius Center for Health Sciences and Primary Care, University Medical Center Utrecht, University Utrecht, Heidelberglaan 100, 3584 CX Utrecht, The Netherlands

**Keywords:** Glioma, Glioblastoma, Symptoms, Adverse events, Toxicity, Patient reported outcomes, PROM

## Abstract

**Background:**

Glioma patients suffer from a wide range of symptoms which influence quality of life negatively. The aim of this review is to give an overview of symptoms most prevalent in glioma patients throughout the total disease trajectory, to be used as a basis for the development of a specific glioma Patient Reported Outcome Measure (PROM) for early assessment and monitoring of symptoms in glioma patients.

**Methods:**

A systematic review focused on symptom prevalence in glioma patients in different phases of disease and treatment was performed in MEDLINE, CINAHL and EMBASE according to PRISMA recommendations. We calculated weighted means for prevalence rates per symptom.

**Results:**

The search identified 2.074 unique papers, of which 32 were included in this review. In total 25 symptoms were identified. The ten most prevalent symptoms were: seizures (37%), cognitive deficits (36%), drowsiness (35%), dysphagia (30%), headache (27%), confusion (27%), aphasia (24%), motor deficits (21%), fatigue (20%) and dyspnea (20%).

**Conclusions:**

Eight out of ten of the most prevalent symptoms in glioma patients are related to the central nervous system and therefore specific for glioma. Our findings emphasize the importance of tailored symptom care for glioma patients and may aid in the development of specific PROMs for glioma patients in different phases of the disease.

**Electronic supplementary material:**

The online version of this article (10.1007/s11060-018-03015-9) contains supplementary material, which is available to authorized users.

## Introduction

Gliomas are the most common primary malignant brain tumors in adults. The annual incidence of malignant glioma in the United States is ~ 5/100,000 with a slight predominance in males [1]. Despite multimodal treatment prognosis remains poor, especially for glioblastoma [2]. Glioma patients often suffer from a wide range of symptoms. These symptoms are often of a neurological nature [3] with a great impact on the patients’ quality of life [4, 5]. Symptom burden in cancer patients may also influence treatment intensity [6]. Improving symptom management in order to maintain quality of life has therefore become a major treatment goal [7].

Symptoms in glioma patients can be caused by the tumor or occur as side effect of treatment. Adequate symptom management for glioma patients relies on knowledge about the prevalence of symptoms in this patient population and efficacy of symptom-aimed treatments [4, 8]. Different papers have reviewed the prevalence or treatment of unique symptoms in glioma patients, such as cognitive deficits [9], seizures [10], and depression [11]. In other papers side effects for specific treatment regimens were reviewed, e.g. toxicity of systemic treatment [12]. However, to our knowledge a review of the symptom burden of the glioma population for the total disease trajectory has not been published.

A thorough overview of symptoms in the total trajectory of glioma patients may also stimulate the development of Patient Reported Outcome Measurements (PROMS) about symptoms for this population. PROMS for assessment of symptoms have been successfully introduced in patient care in the last decade and have been identified as an essential part of symptom management for glioma patients [13–15]. While a few PROMS have been validated to measure symptoms in brain tumor patients (Functional Assessment of Cancer Therapy-Brain/FACT-Br [16], EORTC QLQ-BN20 [17], and MD Anderson Symptom Inventory-Brain/MDASI-BT) [18], only the MDASI-BT is suitable for daily use. The Edmonton Symptom Assessment System (ESAS) is one of the most used PROM’s in symptom care worldwide and has been validated in different groups of patients [19]. Use of this tool resulted in significant improvement of patients symptom burden and symptom management delivered in a diversity of health care settings [20, 21]. However, the ESAS is based on most prevalent symptoms in cancer patients in general and does not include symptoms for specific tumor types like glioma. It has been recommended to add additional questions for specific patient groups [19].

The aim of this study is to perform a systematic review of symptom prevalence in patients with a glioma throughout the total disease trajectory, in order to enhance professionals’ awareness of the symptom burden of glioma patients, and to provide a basis for the development of a symptom-directed glioma PROM suitable for use in clinical practice as well as in research.

## Methods

We performed a systematic literature review using the databases MEDLINE, EMBASE and CINAHL, searching from January 1st 2000 until December 31, 2017. The search domain included synonyms for the ‘glioma’ population and for ‘symptoms, signs, side effects and adverse events’ (see Supplementary Material I). Papers in English or Dutch language were included if they described the prevalence of symptoms, signs or adverse events in adult glioma patients, present in any stage of the disease. We only included papers with 50 patients or more to avoid bias due to small sample sizes. Papers on HRQoL were included when prevalence of symptoms was reported. Papers were excluded if they:

- did not describe original studies
- described only severity of symptoms or hematological toxicities.

Two researchers (FYFdV and MIJ) selected papers based on title and abstract. Agreement about the selection of full papers was reached in consensus meetings. All data from the selected studies by researcher one (FYFdV or MIJ) were checked by researcher two (FYFdV or MIJ). We hand-searched included papers for cross-references. Included studies were evaluated according to the STROBE statement (Strengthening the Reporting of Observational Studies in Epidemiology) [22], see Supplementary Material Table II. We registered symptom prevalence for different phases of disease: at diagnosis; during treatment and follow-up; and in the end-of-life stage. Prevalence of symptoms by glioma grade was also described, when available. For symptoms that were defined differently in the included studies (e.g. cognitive disorders) the most deployed definition was used in this review, but all original descriptions were registered.

For all studies both the characteristics of the study population and the prevalence rates of symptoms were registered for the total group and for subgroups, if available. In one study the first author was contacted to provide additional information about prevalence rates of symptoms not explicitly mentioned in the paper [23].

This systematic review was conducted following the PRISMA statement (Preferred Reporting Items for Systematic reviews and Meta-Analysis) [24].

### Data analysis

We registered the prevalence rates of symptoms per study. Weighted means were calculated per symptom for the total disease trajectory and per phase of disease. Only studies describing the specific symptom were included in this analysis. For symptoms registered separately such as ‘nausea’ and ‘vomiting’ instead of ‘nausea/vomiting’ the highest rates were used for calculating weighted means to achieve prevalence rates best representing the total group. If symptom prevalence was only registered for different phases such as ‘presenting symptoms’ and ‘phase of follow-up’, with no registration of prevalence for the total disease trajectory, we also used the highest reported rates to calculate weighted means.

## Results

### Published papers

**Fig. 1:**
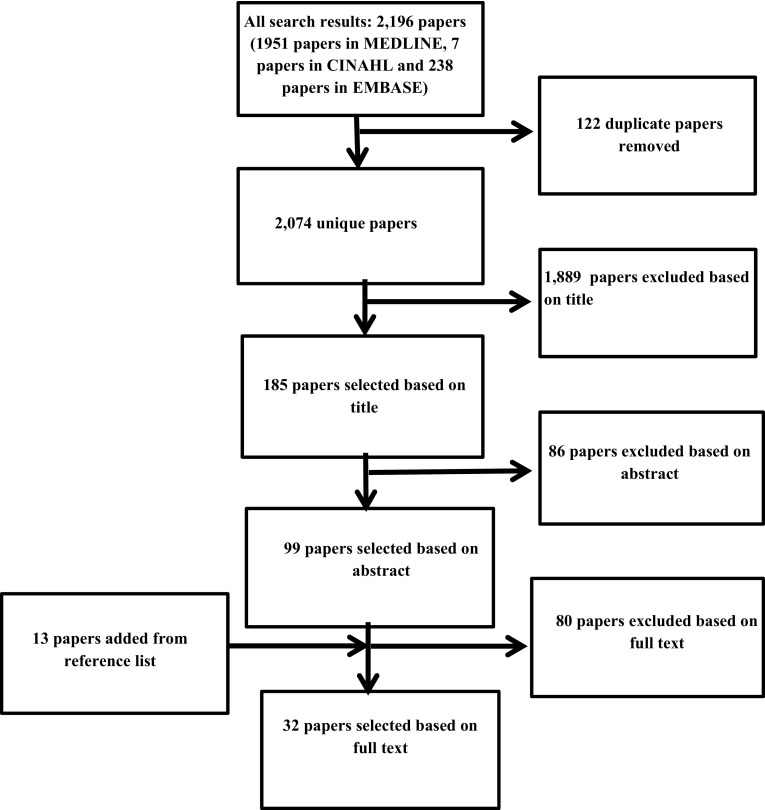
Selection of papers

The search strategy identified 2074 unique papers of which 32 papers were included for this review with a total of 7656 patients included (see Fig. 1).

### Study and patient characteristics

Study characteristics are presented in Table 1. Seven papers used a prospective design [25–31], one of which was a randomized controlled trial [28]. Data were usually collected by a search in the patients’ medical records. In seven studies describing symptoms in the treatment phase, symptoms were registered according to the CTCAE (Common Terminology Criteria for Adverse Events), varying from registering all grades, to only registering grade 3 and 4 [25, 28, 31–35]. In four studies data were collected by means of validated PROMs including symptoms: the EORTC module for brain cancer patients (EORTC QLQ-BN20) [27], the ESAS-r (ESAS revised) [30], the Hospital Anxiety and Depression Scale (HADS) [26, 36], the Fatigue Severity Scale (FSS) [36], and the Epworth Sleepiness Scale (ESS) [36]. Telephone interviews with patients were performed in the study of Sizoo, including 58 patients, in addition to data that were obtained from the medical records [37]. The study of Russo, including 527 patients, used face to face interviews [38]. Questionnaires completed by proxies and physicians after the patient died were conducted in the study of Koekkoek, including 178 patients [23].

**Table 1:** Study specifics

Study	n	Goal	Treatment	Time point	Retrospective/Prospective	Datacollection	References
Bae, 2013	300	Investigate signs and symptoms during temozolomide	Chemotherapy	Treatment	R (cohort)	Medical records on CTCAE version 3.0, grade 1–4	[32]
Brada, 2001	138	Investigate efficacy and toxicity of temozolomide in glioblastoma patients	Chemotherapy	Treatment	P (phase II trial)	Medical records on CTCAE, grade 1–4	[25]
Cao, 2012	112	Investigate safety and efficacy during chemoradiation vs. radiation in elderly patients	Chemoradiation, radiation (hyofractioned) chemotherapy	Diagnosis, treatment	R (cohort)	Medical records on CTCAE version 3.0, grade 1–5	[33]
Chen, 2017	712	Investigate mutant IDH1 and seizures in glioma patients		Diagnosis	R (cross-sectional)	Medical records	[39]
Diamond, 2017	50	Investigate prognostic awareness, communication and cognitive function in patients with glioma		All	P	HADS (score 9 or higher)	[26]
Ening, 2015	233	Investigate risk factors for glioma therapy complications at diagnosis	Surgery, chemotherapy, chemoradiation, radiation	Treatment	R (cohort)	Medical records	[53]
Iuchi, 2014	121	Investigate incidence epilepsy in glioma patients	Surgery, chemoradiation	Diagnosis, FU**	R (cohort)	Medical records	[40]
Jakola, 2012	55	Investigate the association between location, survival, and long-term health in patients with low grade glioma	Surgery, radiation and/or chemotherapy	FU	P	EORTC-BN20 (Likert score 3 and 4)	[27]
Kerkhof, 2013	291	Investigate seizure control of valproic acid	Anti-epileptics	Diagnosis, All (diagnosis and FU)	R (cohort)	Medical records*	[41]
Kim, 2013	406	Investigate incidence epilepsy in glioma patients	Surgery, chemoradation, chemotherapy, radiation	Diagnosis, All (diagnosis and FU)	R (cohort)	Medical records*	[42]
Kocher, 2005	81	Investigate signs and symptoms during chemoradiation	Chemoradiation	Treatment	R (cohort)	Medical records*	[54]
Koekkoek, 2014	178	Investigate signs and symptoms at end-of life	Palliative care	End-of-life	R (cross-sectional)	Developed symptom questionnaire, completed by physician’s and proxies after patient died	[23]
Liang, 2016	184	Investigate indidence of epilepsy in supratentorial glioblastoma patients	Surgery, chemotherapy, (intra-tumor) radiotherapy	Diagnosis, FU	R (cohort)	Medical records	[43]
Malström, 2012	291	Investigate safety and efficacy during chemotherapy vs. radiation in elderly patients	Chemotherapy, (hypofractioned) radiation	Treatment	P (RCT)	WHO grading system for AE grade 2–5; N/V by National Cancer Institute CTC version 2.0	[28]
Mamo, 2017	64	Investigate adverse events in glioblastoma patients with bevacizumab	Targeted therapy	Treatment	R (cohort)	Medical records, CTCAE grade 3 and 4	[34]
Piribauer, 2003	103	Investigate feasibility and toxicity during lomustine therapy in eldery patients	Chemotherapy	Diagnosis	R (cohort)	Medical records	[44]
Posti, 2015	142	Investigate presenting symptoms at diagnosis		Diagnosis	R (cohort)	Medical records from emergency rooms, intensive care unit, and different inpatient wards; hospital and imaging referrals, disch letters	[45]
Rasmussen, 2017	1930	Investigate symptoms in glioma patients	Surgery	Diagnosis	P (cohort)	Danish Neuro-oncology Registry	[29]
Russo, 2017	527	Investigate prevalence of headache in glioma patients		Diagnosis	R (cross-sectional)	Face to face interviews	[38]
Sagberg, 2013	164	Investigate responsiveness of EQ-5D in glioma patients with surgery	Surgery	Diagnosis	R (cross-sectional)	Medical records	[46]
Saito, 2014	76	Investigate signs and symptoms during chemoradiation in eldery patients	Chemoradiation, radiation, chemotherapy	Treatment	R (cohort)	Medical records-CTCAE grade 3 and 4	[35]
Salmaggi, 2005	134	Set up a registry for glioblastoma patients in Lombardia, Italy	Surgery radiation chemotherapy	Diagnosis	R (cohort)	Medical records-reports on signs/symptoms and seizures	[47]
Sanai, 2012	119	Investigate surgery associated complications	Surgery	Diagnosis, treatment	R (cohort)	Medical records and telephone interviews	[48]
Seekatz, 2017	54	Screening for symptom burden in glioma patients		All	P(cohort)	Revised Edmonton Symptom Assessment System (ESAS-r) Score 4–10	[30]
Sizoo, 2010	58	Investigate signs and symptoms at end-of life	Palliative care	End-of-life	R (cohort)	Medical records& charts of nurse specialist on telephone interviews about symptoms based on self-developed checklist	[37]
Stupp, 2002	64	Investigate toxicity of chemoradation	Chemoradiation plus adjuvant chemotharapy	Treatment	P (cohort)	Medical records - CTCAE version 2.0, grade 3–4	[31]
Thrier, 2015	57	Investigate signs and symptoms at end-of life	Palliative care	End-of-life	R (cohort)	Daily reporting of signs and symptoms by standardized protocol	[55]
Valko, 2014	65	Investigate incidence fatigue after surgery in glioma patients	Surgery	Treatment	P (cohort)	Fatigue Severity Scale (FSS score 4–9), Epworth Sleepiness Scale (ESS score 10 or higher), Hospital Anxiety and Depression Scale (HADS score 10 or higher)	[36]
Van Breemen, 2009	108	Investigate seizure control of anti-epileptics	Anti-epileptics	Diagnosis, All (diagnosis and FU)	R (cohort)	Medical records	[49]
Woo, 2014	198	Investigate risk factors for seizures in glioma patients	Surgery, chemoradiation, chemotherapy	Diagnosis, FU	R (cohort)	Medical records	[50]
You, 2012	508	Investigate incidence epilepsy and postoperative seizure control	Surgery	Diagnosis, FU	R (cohort)	Medical records*	[51]
Yuile, 2006	133	Investigate signs and symptoms during radiotherapy	Radiation	Diagnosis	R (cohort)	Medical records	[52]

*Not explicitly mentioned, **FU follow up

Seventeen papers described symptoms in glioma patients at time of diagnosis [29, 33, 38–52]. In sixteen papers symptoms are described in the phase of treatment or follow-up [25, 27, 28, 31–36, 40, 43, 48, 50, 51, 53, 54]. After initial surgery, patients were treated with chemoradiation, chemotherapy or targeted therapy, or radiation. In eleven of the twelve papers recording symptoms and toxicities during or after systemic treatment, chemotherapy or chemoradiation with temozolomide was part of the treatment [25, 28, 31–35, 40, 43, 53, 54]. One paper that registered symptoms during follow-up did not describe which chemotherapy was administered to patients [27]. Three papers described symptoms in the first 10 weeks after surgery: 1–6 weeks postoperatively [48], within 30 days postoperatively [53] and within 10 weeks postoperatively [36]. Symptoms in the end-of-life phase were described in three papers, in which the definition of end of life varied from the moment no next lines of established tumor treatment were possible [37] to 3 months and 1 week before death (retrospectively described by proxies and physicians after the patients’ death) [23], and the last 10 days of life [55]. Two papers registered symptoms in all phases of the disease [26, 30]. Of all papers, nine recorded three or less predefined symptoms: seizures only in seven studies [39–41, 43, 49–51]; and seizures, cognitive deficits and headache in two studies [29, 38].

Patient characteristics are described in Table 2. Most patients were male (60%) and suffered from glioblastoma WHO grade IV.

**Table 2:** Patient characteristics

Study	N	M/F	Age (year) Mean range	KPS (%) (Mean)	KPS ≥ 70%	Glioma WHO II (n)	Glioma WHO III (n)	Glioma WHO IV (n)	Median OS (months) range	References
Bae, 2013	300	187/113	49 17–84	87		20	67	213		[32]
Brada, 2001	138	85/53	54 24–77		100% (KPS > 70%)			138		[25]
Cao, 2012	112	73/39	70 60–86	80		0	0	112	7	[33]
Chen, 2017	712	400/312	55			77	128	507		[39]
Diamond, 2017	50	34/16	50 18–77				16	34		[26]
Ening, 2015	233	117/116	58		79% (KPS > 70%)	0	0	233	9.5 0–72	[53]
Iuchi, 2014	121	74/47	58			19	21	81		[40]
Jakola, 2012	55	30/25	41		91% (KPS ≥ 80%)		55			[27]
Kerkhof, 2013	291	169/122	60 24–85			0	0	291	13	[41]
Kim, 2013	406	244/162	51 18–86		75% (KPS > 70%)	0	124	282		[42]
Kocher, 2005	81	53/28	52 15–72	83		12	22	47		[54]
Koekkoek, 2014	178	125/53	60		20%3 m 2%1 w	0	19	159	12.4 gr III 10.6–14.1 10.6 gr IV 9.2–12.1	[23]
Liang, 2016	184	100/84	49 20–69	47 e 56 we				184		[43]
Malström, 2012	291	173/118	70			0	0	291	8.3 chemo 6.0 rt 7.5 hypofr rt	[28]
Mamo, 2017	64	40/24	54 26–83		88%			64		[34]
Piribauer, 2003	103	65/38	> 55 55–83	79		0	0	103	17.5 py 8.6 pe	[44]
Posti, 2015	142	76/66	60			29	31	82		[45]
Rasmussen, 2017	1930	1158/772	18–79			247	279	1364		[29]
Russo, 2017	527	314/213	53			139	87	268		[38]
Sagberg, 2013	164		56	73		43		121		[46]
Saito, 2014	76	50/26	47 py 71 pe		82% py 70% pe	0	0	76	15.2 12.9–18.5 21.6 py 15.6 pe	[35]
Salmaggi, 2005	134	82/52	61		85%	0	0	134		[47]
Sanai, 2012	119		45 18–81	75		34	23	62		[48]
Seekatz, 2017	54		60 24–79					54		[30]
Sizoo, 2010	58	39/19	52 18–81			0	15	41	21 gr III 11–86 12 gr IV 0.5–71	[37]
Stupp, 2002	64	39/25	52 24–70		64% (KPS > 80%)			64	23	[31]
Thrier, 2015	57	39/18	59	30		0	0	57	12	[55]
Valko, 2014	65	44/21	57	80		0	0	65		[36]
Van Breemen, 2009	108	54/54	40 53			33	75		> 8 years HGG 19 LGG	[49]
Woo, 2014	198	122/76	55 18–88		81%		125	73	9.0 11.0 gr III 8.0 gr IV	[50]
You, 2012	508	306/202	38 16–72		88% (KPS ≥ 80%)	508	0	0	32.9 12–58.3	[51]
Yuile, 2006	133	84/49	59 22–86			0	0	133	10 0.1–51.8	[52]

*Chemo* chemotherapy, *e* with epilepsy, *gr III* grade III glioma, *gr IV* grade IV glioma, *HGG* high grade glioma, *hypofr* hypofractioned, *LGG* low grade glioma, *OS* overal survival rate, *pe* patients of 65 years or older, *py* patients younger than 65 years, *rt* radiotherapy, *we* without epilepsy, *3 m* 3 months before death, *1 w* 1 week before death

### Symptom prevalence throughout the disease course

A total of 25 symptoms were identified: alopecia, anorexia, aphasia, anxiety/depression, cognitive deficits, constipation, confusion, diarrhea, dizziness, drowsiness, dyspepsia, dysphagia, dyspnea, fatigue, gait disturbance, headache, motor deficits, nausea/vomiting, pain, right-left-confusion, seizures, sensory deficits, skin problems, urinary incontinence, and visual deficits. The symptoms nausea/vomiting and anxiety/depression were commonly registered as paired symptoms. In this review we used this paired definition for these symptoms, but if prevalence rates were only described for the symptoms separately in studies, we registered both of these rates.

### Most prevalent symptoms

The prevalence of symptoms for the total disease trajectory is recorded in Supplementary Material Table III. Table 3 shows weighted means of symptom prevalence. The ten most prevalent symptoms for the total disease trajectory are: seizures (37%), cognitive deficits (36%), drowsiness (35%), dysphagia (30%), headache (27%), confusion (27%), aphasia (24%), motor deficits (21%), fatigue (20%) and dyspnea (20%).

**Table 3:** Weighted means (in %) of symptom prevalence

	Seizures (1)	Cognitive deficits (2)	Drowsiness (3)	Dysphagia	Headache	Confusion(4)	Aphasia (5)	Motor deficits (6)	Fatigue (7)	Dyspnea (8)	Nausea/vomiting (9)	Urinary incontinence (10)	Pain (11)	Anxiety/depression (12)	Anorexia (13)	Sensory deficits	Dizziness (14)	Visual deficits (15)	Gait disturbance	Alopecia	Skin problems (16)	Right left confusion	Constipation	Diarrhea	Dyspepsia
Total disease trajectory	36.5	35.9	35.3	30.0	27.2	26.5	23.7	21.4	20.3	19.6	19.0	16.5	15.5	15.1	13.5	13.3	13.0	12.1	10.0	8.1	6.7	5.0	4.3	2.6	2.0
Diagnostic phase	34.7	36.0	15.0	4.0	30.5		20.1	21.6			6.5					13.3	23.5	6.7	10.0						
Treatment/FU phase	36.7	18.3	7.7		7.7	3.0	8.4	10.5	13.7		23.2	7.5		6.8	12.7		5.9	12.8		8.1	5.2	5.0	3.8	2.6	2.0
End-of-Life phase	44.6	44.3	81.3	41.9	37.3	40.3	48.0	44.2	49.9	17.7	19.2	37.0	15.2	15.8			2.0	23.0			22.0		9.0		

The symptoms presented here as most prevalent are not necessarily the symptoms reported in most studies. Confusion and dyspnea for example are reported in only three studies, including two studies in the end of life phase [23, 37]. When excluding studies which registered only unique symptoms (n = 9), the most frequently reported symptoms in the 23 remaining studies are: seizures (16 studies), headache (14 studies), fatigue (13 studies), nausea/vomiting (12 studies), and motor deficits (10 studies).

### Symptom prevalence per phase

The prevalence of symptoms per phase of disease is also recorded in Supplementary Material Table III, and weighted means in Table 3. The five most prevalent symptoms in the diagnostic phase are cognitive deficits (36%), seizures (35%), headache (31%), dizziness (24%), and motor deficits (22%). In the treatment and follow-up phase the most prevalent symptoms are seizures (37%), nausea/vomiting (23%), cognitive deficits (18%), fatigue (14%), visual deficits (13%) and anorexia (13%). Nausea/vomiting is more prevalent during systemic treatment than postoperatively. Other symptoms in the treatment phase are less common, with weighted prevalence means of 10% or less. In the end-of-life phase, drowsiness (81%), fatigue (50%), aphasia (48%), seizures (45%), cognitive deficits (44%), and motor deficits (44%) are most prevalent.

Most of the 25 symptoms are described in all three phases of disease and treatment. Alopecia, anorexia, dyspepsia and diarrhea are only reported during systemic treatment or radiation.

### Symptom prevalence by tumor grade

In some studies symptom prevalence was described by tumor grade (see Table 4). Seizures show a high prevalence in all grades. Cognitive disorders are more prevalent in grade III and IV tumors, but their prevalence in grade II tumors is still considerable. The prevalence of headache is less different between tumor grades (22–38%).

**Table 4:** Symptoms by grade of glioma

Study	Histological grade glioma		
	WHO II	WHO III	WHO IV
Seizures			
Iuchi	47% pr 74% t	29% pr 67% t	20% pr 57% t
Kim		34–37%	29%
Posti	83%	65%	38%
Van Breemen	70% pr 76% t		52% pr 80% t
Rasmussen	58% pr	45% pr	24%
Cognitive disorders			
Posti	21%	45%	74%
Rasmussen	24%	41%	48%
Headache			
Rasmussen	22%	30%	38%

## Discussion

The most prevalent symptoms in patients with glioma throughout the total disease trajectory in this review are seizures, cognitive deficits, drowsiness, dysphagia, headache, confusion, aphasia, motor deficits, fatigue, and dyspnea. The exact prevalence of symptoms varies strongly between different phases of the disease. The findings of the review emphasize the unique nature of glioma patients’ symptom burden, which is closer related to the symptoms of a brain disease than to the symptom burden of cancer patients in general [56, 57].

Seizures are highly prevalent in glioma patients. Seizures were assessed frequently and were registered exclusively in seven papers [39–41, 43, 49–51]. To avoid bias of increased attention for this symptom in these papers, we also calculated weighted mean prevalence of seizures in papers not exclusively registering the symptom. The prevalence of seizures then decreased to 28%, which is still high. The symptoms confusion, dysphagia and dyspnea show especially high prevalence in the end-of-life phase, but are reported less frequently during the phases of diagnosis and treatment and follow-up.

This review shows the unique nature of glioma patients’ symptom burden. Symptoms seem to be largely caused by the tumor itself and to a much lesser degree by treatment. This is confirmed by results of other studies. A review of Sizoo [58] about symptoms in the end-of life-phase for glioma patients showed a comparable or even higher prevalence of neurological symptoms such as seizures, cognitive decline and progressive neurological deficits compared to our study. Except for fatigue, the more generally acknowledged end-of-life symptoms in cancer such as anorexia and weight loss occur less often in glioma patients than in other groups of palliative care patients. Ostgathe concluded that the prevalence of confusion in the end-of-life phase was significantly higher in patients with primary brain tumors than in patients with brain metastases or a general palliative care population [59]. In a systematic review of Wei [12] reporting toxicities in patients with high grade glioma treated with chemo-radiation, gastrointestinal toxicities and fatigue remained under 7%.

Eight of the ten most prevalent symptoms in this review are included in at least two of the three existing PROMS measuring symptoms in glioma patients with the same or different wordings (EORTC QLQ-BN20, FACT-Br, MDASI-BT). Confusion and dysphagia are not included in one of them. This could be because of their prominence in the end-of-life phase: other PROMS did not include all phases of the total disease trajectory in development of the PROM. Dyspnea and fatigue are reported in the core versions of the three PROMS (dyspnea not on the FACT-Br). ‘Visual deficits’ is included in all three mentioned PROMS, but showed a prevalence of only 12% in this review. No other neurological symptoms are included in at least two of those three PROMS.

### Limitations

In this review only seven of the 32 studies we included used prospective data. In only four studies patients were asked about symptoms themselves by a validated PROM, only one of which was specifically developed for patients with brain tumors (QLQ-BN-20). Most studies used collected data in medical records only, which possibly resulted in symptoms being missed because patients were not asked about them or the symptoms were not documented in the records. Patients are more likely to reveal their real symptom burden with the use of a questionnaire than through spontaneous self-report [60]. This phenomenon is likely to have led to underreporting of symptoms. The poor representation of brain tumor PROMS in this review is likely to be caused by difficulties in using these questionnaires in this patient population in general: questionnaires are quickly experienced as being too long or difficult due to cognitive or functional impairments, which can result in decreased compliance and use [13]. A glioma PROM that is perceived as brief and easy could increase its use. Secondly, we had to exclude some studies who did use a specific PROM but only reported scale scores, and not prevalence. Another limitation of this review is the use of different definitions for symptoms and pairing of symptoms in the included studies, which may have influenced our results.

### Strengths

This is the first published systematic review of symptoms in glioma patients throughout the whole continuum of the disease trajectory, as well as per phase and (where possible) by grade of glioma.

### Conclusion and recommendations

Eight out of ten of the most prevalent symptoms in glioma patients in this review are neurological in nature. Because of this unique symptom burden differing from symptoms in cancer patients in general and its effect on quality of life and treatment, the results of our review stress a need for tailored symptom care in glioma patients. This care will be improved by use of a specific glioma PROM focusing on glioma specific symptoms throughout all disease stages and suitable for daily use.

## Electronic supplementary material

Below is the link to the electronic supplementary material.

Supplementary material 1 (DOCX 13 KB)

Supplementary material 2 (DOCX 20 KB)

Supplementary material 3 (DOCX 63 KB)
