# Supplementary material for: Prevalence of symptoms in glioma patients throughout the disease trajectory: a systematic review
Source: J Neurooncol. 2018 Oct 30;140(3):485–96. doi: 10.1007/s11060-018-03015-9 (PMC6267240; doi:10.1007/s11060-018-03015-9)
Supplement: Supplementary file 1 — Supplementary material 1 (DOCX 13 KB) [file 11060_2018_3015_MOESM1_ESM.docx]

**Supplementary I: Search strategy**

**MEDLINE: 1951 hits**

((((((((glioma[Title] OR astrocytoma[Title] OR glioblastoma[Title] OR lgg[Title] OR hgg[Title] OR gbm[Title] OR glioma[MeSH])) AND (full text[sb] AND ( "2000/01/01"[PDat] : "2017/12/31"[PDat] ) AND ( Dutch[lang] OR English[lang] ) ))) AND (((symptom[Title] OR symptoms[Title] OR sign[Title] OR signs[Title] OR adverse event[Title] OR adverse events[Title] OR adverse effect[Title] OR adverse effects[Title] OR toxicity[Title] OR toxicities[Title]) OR ("Signs and Symptoms"[MeSH] OR "Symptom Assessment"[MeSH] OR "Drug-Related Side Effects and Adverse Reactions"[MeSH])) AND (full text[sb] AND ( "2000/01/01"[PDat] : "2017/12/31"[PDat] ) AND ( Dutch[lang] OR English[lang] ) ))) AND (full text[sb] AND ( "2000/01/01"[PDat] : "2017/12/31"[PDat] ) AND ( Dutch[lang] OR English[lang] ) ))) AND (full text[sb] AND ( "2000/01/01"[PDat] : "2017/12/31"[PDat] ) AND ( Dutch[lang] OR English[lang] ) ))

**EMBASE: 238 hits (exluding MEDLINE 120 hits)**

('glioma':ti OR 'glioblastoma':ti OR 'astrocytoma':ti OR 'hgg':ti OR 'lgg':ti OR 'gbm':ti) AND ('symptom':ti OR 'symptoms':ti OR 'sign':ti OR 'signs':ti OR 'adverse event':ti OR 'adverse events':ti OR 'adverse effect':ti OR 'adverse effets':ti OR 'toxicity':ti OR 'toxicities':ti) AND ([dutch]/lim OR [english]/lim) AND [2000-2017]/py

**CINAHL: 7 hits (excluding MEDLINE 3 hits)**

(MJ glioma OR TI glioma OR TI glioblastoma OR TI astrocytoma OR TI hgg OR TI lgg OR TI gbm) AND (MJ symptoms OR TI symptom OR TI symptoms OR TI sign OR TI signs OR TI adverse event OR TI adverse events OR TI adverse effect OR TI adverse effects OR TI toxicity OR TI toxicties)

Limits: link to full text, language: English, publication date: 01/01/2000 – 12/31/2017
