# Supplementary material for: Prevalence of symptoms in glioma patients throughout the disease trajectory: a systematic review
Source: J Neurooncol. 2018 Oct 30;140(3):485–96. doi: 10.1007/s11060-018-03015-9 (PMC6267240; doi:10.1007/s11060-018-03015-9)
Supplement: Supplementary file 2 — Supplementary material 2 (DOCX 20 KB) [file 11060_2018_3015_MOESM2_ESM.docx]

**SUPPLEMENTARY II: STROBE STATEMENT CHECKLIST**

| Study→  Itemnr↓ | Bae | Brada | Cao | Chen | Diamond | Ening | Iuchi | Jakola | Kerkhof | Kim | Kocher | Koekkoek | Liang | Malstrom | Mamo | Piribauer | Posti | Rasmussen | Russo | Sagberg | Saito | Salmaggi | Sanai | Seekatz | Sizoo | Stupp | Thrier | Valko | VanBreemen | Woo | You | Yuile |
| --- | --- | --- | --- | --- | --- | --- | --- | --- | --- | --- | --- | --- | --- | --- | --- | --- | --- | --- | --- | --- | --- | --- | --- | --- | --- | --- | --- | --- | --- | --- | --- | --- |
| 1 | x | x | x | x | x | x | x | x | x | x | x | x | x | x | x | x | x | x | x | x | x | x | x | x | x | x | x | x | x | x | x | x |
| 2 | x | x | x | x | x | x | x | x | x | x | x | x | x | x | x | x | x | x | x | x | x | x | x | x | x | x | x | x | x | x | x | x |
| 3 | x | x | x | x | x | x | x | x | x | x | x | x | x | x | x | x | x | x | x | x | x | x | x | x | x | x | x | x | x | x | x | x |
| c4 | x | x | x | x | x | x | x | x | x | x | x | x | x | x | x | x | x | x | x | x | x | x | x | x | x | x | x | x | x | x | x | x |
| 5 | x | x | x | x | x | x | x | x | x | x | x | x | x | x | x | x | x | x | x | x | x | x | x | x | x | x | x | x | x | x | x | x |
| 6 | x | x | x | x | x | x | x | x | x | x | x | x | x | x | x | x | x | x | x | x | x | x | x | x | x | x | x | x | x | x | x | x |
| 7 | x | x | x | x | x | x | x | x | x | x | x | x | x | x | x | x | x | x | x | x | x | x | x | x | x | x | x | x | x | x | x | x |
| 8 | x | x | x | x | x | x | x | x | x | x | x | x | x | x | x | x | x | x | x | x | x | x | x | x | x | x | x | x | x | x | x | x |
| 9 | - | - | x | - | - | - | - | x | x | - | - | - | - | - | - | - | - | - | x | - | - | - | x | - | - | - | - | - | - | - | - | - |
| 10 | x | x | x | x | x | x | x | x | x | x | x | x | x | x | x | x | x | x | x | x | x | x | x | x | x | x | x | x | x | x | x | x |
| 11 | x | x | x | x | x | x | x | x | x | x | x | x | x | x | x | x | x | x | x | x | x | x | x | x | x | x | x | x | x | x | x | x |
| 12 | - | x | - | - | - | - | - | - | - | - | - | - | - | - | - | - | - | x | - | x | - | - | x | x | - | - | - | - | - | - | - | - |
| 13 | - | - | - | - |  | - | x | - | - | x | - | - | x | - | x | - | - | - | x | - | - | x | x | x | x | - | - | - | - | - | x | x |
| 14 | x | - | x | - | x | x | x | x | x | x | - | - | - | - | x | x | x | - | x | x | - | x | x | x | x | - | x | x | x | x | x | x |
| 15 | x | x | x | x | x | x | x | x | x | x | x | x | x | x | x | x | x | x | x | x | x | x | x | x | x | x | x | x | x | x | x | x |
| 16 | x | x | x | x | x | x | x | x | x | x | x | x | x | x | x | x | x | x | x | x | x | x | x | x | x | x | x | x | x | x | x | x |
| 17 | x | x | x | x | x | x | x | x | x | x | x | x | x | x | x | x | x | x | x | x | x | x | x | x | x | x | x | x | x | x | x | x |
| 18 | x | x | x | x | x | x | x | x | x | x | x | x | x | x | x | x | x | x | x | x | x | x | x | x | x | x | x | x | x | x | x | x |
| 19 | x | - | x | x | x | - | x | x | x | - | x | x | x | - | x | x | x | x | x | x | - | - | x | x | x | x | x | x | x | x | x | x |
| 20 | x | - | x | x | x | - | x | x | x | x | x | x | x | x | - | x | x | x | x | x | - | - | x | x | x | x | x | x | x | x | x | x |
| 21 | - | - | - | x | - | - | x | - | - | - | - | x | - | x | - | - | x | x | x | - | - | - | x | - | - | - | - | - | - | - | - | - |
| 22 | x | x | x | x | x | - | x | x | x | x | - | x | x | x | - | - | x | x | x | x | x | - | x | - | x | - | x | x | x | x | x | - |

9: Describe any efforts to address potential sources of bias

12c: Explain how missing data were addressed

13b: Give reasons for non-participation at each stage

14b: Indicate number of participants with missing data for each variable of interest

19: Discuss limitations of the study

20: Give a cautious overall interpretation or results

21: Discuss the generalizability

22: Give the source of funding and the role of funders for the present study
