# Supplementary material for: Prevalence of symptoms in glioma patients throughout the disease trajectory: a systematic review
Source: J Neurooncol. 2018 Oct 30;140(3):485–96. doi: 10.1007/s11060-018-03015-9 (PMC6267240; doi:10.1007/s11060-018-03015-9)
Supplement: Supplementary file 3 — Supplementary material 3 (DOCX 63 KB) [file 11060_2018_3015_MOESM3_ESM.docx]

**Supplementary Table III: Prevalence of symptoms (%) per disease phase, data from individual studies**

| Total disease trajectory | | | | | | | | | | | | | | | | | | | | | | | | | | | | | | | | | | | | | | | | | | | | | | | | | | | | | | | |  |
| --- | --- | --- | --- | --- | --- | --- | --- | --- | --- | --- | --- | --- | --- | --- | --- | --- | --- | --- | --- | --- | --- | --- | --- | --- | --- | --- | --- | --- | --- | --- | --- | --- | --- | --- | --- | --- | --- | --- | --- | --- | --- | --- | --- | --- | --- | --- | --- | --- | --- | --- | --- | --- | --- | --- | --- | --- |
| \| Reference \| Seizures (1) \| Cognitive deficits (2) \| Drowsiness (3) \| Dysphagia \| Headache \| Confusion( 4) \| Aphasia (5) \| Motor deficits (6) \| Fatigue (7) \| Dyspnea (8) \| Nausea/ Vomiting (9) \| Urinary incontinence (10) \| Pain (11) \| Anxiety/ Depression (12) \| Anorexia (13) \| Sensory deficits \| Dizziness (14) \| Visual deficits (15) \| Gait disturbance \| Alopecia \| Skin problems (16) \| Right left confusion \| Constipation \| Diarrhea \| Dyspepsia \| \| --- \| --- \| --- \| --- \| --- \| --- \| --- \| --- \| --- \| --- \| --- \| --- \| --- \| --- \| --- \| --- \| --- \| --- \| --- \| --- \| --- \| --- \| --- \| --- \| --- \| --- \| | | | | | | | | | | | | | | | | | | | | | | | | | | | | | | | | | | | | | | | | | | | | | | | | | | | | | | | |  |
| 32 |  | |  | |  | |  | | 11 | |  | |  | |  | | 10 | |  | | 44n  37v | | 4 | |  | |  | | 14 | |  | | | 8 | |  | | |  | | 10 | | 12 | |  | | | 4 | | | 2 | | |  | |  |
| 25 | 2 | |  | | 9 | |  | | 8 | | 3 | |  | | 5 | | 20 | |  | | 30n  28v | |  | |  | |  | | 10 | |  | | | 2 | |  | | |  | | 4 | | 4 | |  | | | 5 | | | 4 | | | 2 | |  |
| 33 | 31pr | | 52pr | |  | |  | | 43pr | |  | |  | |  | |  | |  | |  | |  | |  | |  | |  | |  | | |  | | 7pr | | |  | |  | |  | |  | | |  | | |  | | |  | |  |
| 39 | 39pr | |  | |  | |  | |  | |  | |  | |  | |  | |  | |  | |  | |  | |  | |  | |  | | |  | |  | | |  | |  | |  | |  | | |  | | |  | | |  | |  |
| 26 |  | |  | |  | |  | |  | |  | |  | |  | |  | |  | |  | |  | |  | | 38a  38d | |  | |  | | |  | |  | | |  | |  | |  | |  | | |  | | |  | | |  | |  |
| 53 | 16 | |  | | 3 | |  | | 1 | |  | | 3 | | 10 | | 5 | |  | | 12 | | 12 | |  | | 4 | |  | |  | | |  | |  | | |  | |  | | 1 | |  | | |  | | |  | | |  | |  |
| 40 | 61fu | |  | |  | |  | |  | |  | |  | |  | |  | |  | |  | |  | |  | |  | |  | |  | | |  | |  | | |  | |  | |  | |  | | |  | | |  | | |  | |  |
| 27 |  | | 38 | |  | |  | |  | |  | | 21 | | 36 | | 44 | |  | |  | |  | |  | |  | |  | |  | | |  | | 21 | | |  | |  | |  | |  | | |  | | |  | | |  | |  |
| 41 | 62 | |  | |  | |  | |  | |  | |  | |  | |  | |  | |  | |  | |  | |  | |  | |  | | |  | |  | | |  | |  | |  | |  | | |  | | |  | | |  | |  |
| 42 | 31 | | 16pr | |  | |  | | 35pr | |  | |  | |  | |  | |  | |  | |  | |  | |  | |  | |  | | |  | |  | | |  | |  | |  | |  | | |  | | |  | | |  | |  |
| 54 | 12 | |  | |  | |  | | 14 | |  | |  | | 4 | | 9 | |  | | 41n | |  | |  | |  | |  | |  | | | 5 | |  | | |  | |  | |  | |  | | |  | | |  | | |  | |  |
| 23 | 38m  26w | | 38m  48w | | 31m  75w | | 25w | | 40m  35w | | 24m  44w | | 39m  48w | | 42m  41w | | 58m  58w | | 24w | | 19m,n  16w,n | | 23m  36w | | 10m  12w | | 18m,a  18w,a  12m,d  8w,d | |  | |  | | |  | | 22m  23w | | |  | |  | |  | |  | | |  | | |  | | |  | |  |
| 43 | 69 | |  | |  | |  | |  | |  | |  | |  | |  | |  | |  | |  | |  | |  | |  | |  | | |  | |  | | |  | |  | |  | |  | | |  | | |  | | |  | |  |
| 28 | 10 | |  | |  | |  | |  | |  | |  | |  | | 9 | |  | | 4n  2v | |  | |  | |  | |  | |  | | |  | |  | | |  | |  | |  | |  | | |  | | |  | | |  | |  |
| 34 | 13 | |  | |  | |  | |  | |  | |  | |  | | 25 | |  | |  | |  | |  | |  | |  | |  | | |  | |  | | |  | |  | |  | |  | | |  | | |  | | |  | |  |
| 44 | 27pr | |  | |  | |  | | 43pr | |  | |  | |  | |  | |  | |  | |  | |  | |  | |  | |  | | | 30pr | |  | | |  | |  | |  | |  | | |  | | |  | | |  | |  |
| 45 | 53pr | | 57pr | |  | |  | | 20pr | |  | | 25pr | | 22pr | |  | |  | | 6pr | |  | |  | |  | |  | | 6pr | | | 9pr | | 9pr | | | 10pr | |  | |  | |  | | |  | | |  | | |  | |  |
| 29 | 31pr | | 43pr | |  | |  | | 35pr | |  | |  | |  | |  | |  | |  | |  | |  | |  | |  | |  | | |  | |  | | |  | |  | |  | |  | | |  | | |  | | |  | |  |
| 38 | 9pr | | 20pr | |  | |  | | 13pr | |  | |  | |  | |  | |  | |  | |  | |  | |  | |  | |  | | |  | |  | | |  | |  | |  | |  | | |  | | |  | | |  | |  |
| 46 | 60pr | | 39pr | |  | |  | | 27pr | |  | | 30pr | | 28pr | |  | |  | | 7n,pr | |  | |  | |  | |  | |  | | | 32pr | |  | | |  | |  | |  | |  | | |  | | |  | | |  | |  |
| 35 |  | | 4 | |  | |  | |  | |  | |  | |  | | 3 | |  | |  | |  | |  | |  | |  | |  | | |  | |  | | |  | |  | | 1 | |  | | | 1 | | |  | | |  | |  |
| 47 | 31pr | | 19pr | |  | | 4pr | | 16pr | |  | | 15pr | | 15pr | |  | |  | |  | |  | |  | |  | |  | |  | | |  | | 4pr | | |  | |  | |  | |  | | |  | | |  | | |  | |  |
| 48 | 45pr | |  | |  | |  | |  | |  | | 22pr | |  | |  | |  | |  | |  | |  | |  | |  | | 22pr | | |  | | 9ep | | |  | |  | |  | | 5ep | | |  | | |  | | |  | |  |
| 30 |  | |  | | 59 | |  | |  | |  | |  | |  | | 59 | | 9 | | 4 | |  | | 17 | | 37a  28d | | 20 | |  | | |  | |  | | |  | |  | |  | |  | | |  | | |  | | |  | |  |
| 37 | 45 | | 33 | | 87 | | 71 | | 33 | | 29 | |  | | 51 | | 25 | | 16 | | 20 | | 40 | | 25 | | 9 | |  | |  | | | 2 | |  | | |  | |  | |  | |  | | | 9 | | |  | | |  | |  |
| 31 |  | |  | |  | |  | |  | |  | |  | |  | | 13r  16z | |  | | 11r  16z | |  | |  | |  | |  | |  | | |  | |  | | |  | |  | | 2r  0z | |  | | |  | | |  | | |  | |  |
| 55 | 65 | |  | | 95 | | 65 | | 33 | |  | |  | |  | |  | |  | | 19v | |  | |  | |  | |  | |  | | |  | |  | | |  | |  | | 22 | |  | | |  | | |  | | |  | |  |
| 36 |  | |  | | 22 | |  | |  | |  | |  | |  | | 48 | |  | |  | |  | |  | | 17a  9d | |  | |  | | |  | |  | | |  | |  | |  | |  | | |  | | |  | | |  | |  |
| 49 | 79 | |  | |  | |  | |  | |  | |  | |  | |  | |  | |  | |  | |  | |  | |  | |  | | |  | |  | | |  | |  | |  | |  | | |  | | |  | | |  | |  |
| 50 | 57 | |  | |  | |  | |  | |  | |  | |  | |  | |  | |  | |  | |  | |  | |  | |  | | |  | |  | | |  | |  | |  | |  | | |  | | |  | | |  | |  |
| 51 | 69pr | |  | |  | |  | |  | |  | |  | |  | |  | |  | |  | |  | |  | |  | |  | |  | | |  | |  | | |  | |  | |  | |  | | |  | | |  | | |  | |  |
| 52 | 17pr | |  | | 15pr | |  | | 30pr | |  | | 6pr | | 20pr | |  | |  | |  | |  | |  | |  | |  | |  | | |  | |  | | |  | |  | |  | |  | | |  | | |  | | |  | |  |
| Presenting symptoms | | | | | | | | | | | | | | | | | | | | | | | | | | | | | | | | | | | | | | | | | | | | | | | | | | | | | | | |  |
| \| Reference \| Seizures (1) \| Cognitive deficits (2) \| Drowsiness (3) \| Dysphagia \| Headache \| Confusion( 4) \| Aphasia (5) \| Motor deficits (6) \| Fatigue (7) \| Dyspnea (8) \| Nausea/ Vomiting (9) \| Urinary incontinence (10) \| Pain (11) \| Aniety/De-pression (12) \| Anorexia (13) \| Sensory deficits \| Dizziness (14) \| Visual deficits (15) \| Gait distur-bance \| Alopecia \| Skin problems (16) \| Right left confusion \| Constipation \| Diarrhea \| Dyspepsia \| \| --- \| --- \| --- \| --- \| --- \| --- \| --- \| --- \| --- \| --- \| --- \| --- \| --- \| --- \| --- \| --- \| --- \| --- \| --- \| --- \| --- \| --- \| --- \| --- \| --- \| --- \| | | | | | | | | | | | | | | | | | | | | | | | | | | | | | | | | | | | | | | | | | | | | | | | | | | | | | | | |  |
| 33 | | 31 | | 52 | |  | |  | | 43 | |  | |  | |  | |  | |  | |  | |  | |  | |  | |  | |  | | |  | | | 7 | |  | |  | |  | |  | | |  | | |  | | |  | |
| 39 | | 39 | |  | |  | |  | |  | |  | |  | |  | |  | |  | |  | |  | |  | |  | |  | |  | | |  | | |  | |  | |  | |  | |  | | |  | | |  | | |  | |
| 40 | | 25 | |  | |  | |  | |  | |  | |  | |  | |  | |  | |  | |  | |  | |  | |  | |  | | |  | | |  | |  | |  | |  | |  | | |  | | |  | | |  | |
| 41 | | 42 | |  | |  | |  | |  | |  | |  | |  | |  | |  | |  | |  | |  | |  | |  | |  | | |  | | |  | |  | |  | |  | |  | | |  | | |  | | |  | |
| 42 | | 26 | | 16 | |  | |  | | 35 | |  | |  | |  | |  | |  | |  | |  | |  | |  | |  | |  | | |  | | |  | |  | |  | |  | |  | | |  | | |  | | |  | |
| 43 | | 23 | |  | |  | |  | |  | |  | |  | |  | |  | |  | |  | |  | |  | |  | |  | |  | | |  | | |  | |  | |  | |  | |  | | |  | | |  | | |  | |
| 44 | | 27 | |  | |  | |  | | 43 | |  | |  | |  | |  | |  | |  | |  | |  | |  | |  | |  | | | 30 | | |  | |  | |  | |  | |  | | |  | | |  | | |  | |
| 45 | | 53 | | 57 | |  | |  | | 20 | |  | | 25 | | 22 | |  | |  | | 6 | |  | |  | |  | |  | | 6 | | | 9 | | | 9 | | 10 | |  | |  | |  | | |  | | |  | | |  | |
| 29 | | 31 | | 43 | |  | |  | | 35 | |  | |  | |  | |  | |  | |  | |  | |  | |  | |  | |  | | |  | | |  | |  | |  | |  | |  | | |  | | |  | | |  | |
| 38 | | 9 | | 20 | |  | |  | | 13 | |  | |  | |  | |  | |  | |  | |  | |  | |  | |  | |  | | |  | | |  | |  | |  | |  | |  | | |  | | |  | | |  | |
| 46 | | 60 | | 39 | |  | |  | | 27 | |  | | 30 | | 28 | |  | |  | | 7n | |  | |  | |  | |  | |  | | | 32 | | |  | |  | |  | |  | |  | | |  | | |  | | |  | |
| 47 | | 31 | | 19 | |  | | 4 | | 16 | |  | | 15 | | 15 | |  | |  | |  | |  | |  | |  | |  | |  | | |  | | | 4 | |  | |  | |  | |  | | |  | | |  | | |  | |
| 48 | | 45 | |  | |  | |  | |  | |  | | 22 | |  | |  | |  | |  | |  | |  | |  | |  | | 22 | | |  | | |  | |  | |  | |  | |  | | |  | | |  | | |  | |
| 49 | | 57 | |  | |  | |  | |  | |  | |  | |  | |  | |  | |  | |  | |  | |  | |  | |  | | |  | | |  | |  | |  | |  | |  | | |  | | |  | | |  | |
| 50 | | 27 | |  | |  | |  | |  | |  | |  | |  | |  | |  | |  | |  | |  | |  | |  | |  | | |  | | |  | |  | |  | |  | |  | | |  | | |  | | |  | |
| 51 | | 69 | |  | |  | |  | |  | |  | |  | |  | |  | |  | |  | |  | |  | |  | |  | |  | | |  | | |  | |  | |  | |  | |  | | |  | | |  | | |  | |
| 52 | | 17 | |  | | 15 | |  | | 30 | |  | | 6 | | 20 | |  | |  | |  | |  | |  | |  | |  | |  | | |  | | |  | |  | |  | |  | |  | | |  | | |  | | |  | |
| During treatment and follow-up | | | | | | | | | | | | | | | | | | | | | | | | | | | | | | | | | | | | | | | | | | | | | | | | | | | | | | | |  |
| \| Reference \| Seizures (1) \| Cognitive deficits (2) \| Drowsiness (3) \| Dysphagia \| Headache \| Confusion( 4) \| Aphasia (5) \| Motor deficits (6) \| Fatigue (7) \| Dyspnea (8) \| Nausea/ Vomiting (9) \| Urinary incontinence (10) \| Pain (11) \| Aniety/De-pression (12) \| Anorexia (13) \| Sensory deficits \| Dizziness (14) \| Visual deficits (15) \| Gait distur-bance \| Alopecia \| Skin problems (16) \| Right left confusion \| Constipation \| Diarrhea \| Dyspepsia \| \| --- \| --- \| --- \| --- \| --- \| --- \| --- \| --- \| --- \| --- \| --- \| --- \| --- \| --- \| --- \| --- \| --- \| --- \| --- \| --- \| --- \| --- \| --- \| --- \| --- \| --- \| | | | | | | | | | | | | | | | | | | | | | | | | | | | | | | | | | | | | | | | | | | | | | | | | | | | | | | | |  |
| 32 | |  | |  | |  | |  | | 11 | |  | |  | |  | | 10 | |  | | 44n  37v | | 4 | |  | |  | | 14 | | |  | 8 | | |  | | |  | | 10 | | 12 | | |  | | | 4 | | | 2 | |  | |
| 25 | | 2 | |  | | 9 | |  | | 8 | | 3 | |  | | 5 | | 20 | |  | | 30n  28v | |  | |  | |  | | 10 | | |  | 2 | | |  | | |  | | 4 | | 4 | | |  | | | 5 | | | 4 | | 2 | |
| 26 | |  | |  | |  | |  | |  | |  | |  | |  | |  | |  | |  | |  | |  | | 38a  38d | |  | | |  |  | | |  | | |  | |  | |  | | |  | | |  | | |  | |  | |
| 53 | | 16 | |  | | 3 | |  | | 1 | |  | | 3 | | 10 | | 5 | |  | | 12 | | 12 | |  | | 4 | |  | | |  |  | | |  | | |  | |  | | 1 | | |  | | |  | | |  | |  | |
| 40 | | 61fu | |  | |  | |  | |  | |  | |  | |  | |  | |  | |  | |  | |  | |  | |  | | |  |  | | |  | | |  | |  | |  | | |  | | |  | | |  | |  | |
| 27 | |  | | 38 | |  | |  | |  | |  | | 21 | | 36 | | 44 | |  | |  | |  | |  | |  | |  | | |  |  | | | 21 | | |  | |  | |  | | |  | | |  | | |  | |  | |
| 54 | | 12 | |  | |  | |  | | 14 | |  | |  | | 4 | | 9 | |  | | 41n | |  | |  | |  | |  | | |  | 5 | | |  | | |  | |  | |  | | |  | | |  | | |  | |  | |
| 43 | | 45fu | |  | |  | |  | |  | |  | |  | |  | |  | |  | |  | |  | |  | |  | |  | | |  |  | | |  | | |  | |  | |  | | |  | | |  | | |  | |  | |
| 28 | | 10 | |  | |  | |  | |  | |  | |  | |  | | 9 | |  | | 4n  2v | |  | |  | |  | |  | | |  |  | | |  | | |  | |  | |  | | |  | | |  | | |  | |  | |
| 34 | | 13 | |  | |  | |  | |  | |  | |  | |  | | 25 | |  | |  | |  | |  | |  | |  | | |  |  | | |  | | |  | |  | |  | | |  | | |  | | |  | |  | |
| 35 | |  | | 4 | |  | |  | |  | |  | |  | |  | | 3 | |  | |  | |  | |  | |  | |  | | |  |  | | |  | | |  | |  | | 1 | | |  | | | 1 | | |  | |  | |
| 48 | |  | |  | |  | |  | |  | |  | | 13ep  8,6m | |  | |  | |  | |  | |  | |  | |  | |  | | |  |  | | | 9ep  7,6m | | |  | |  | |  | | | 5ep  3,6m | | |  | | |  | |  | |
| 31 | |  | |  | |  | |  | |  | |  | |  | |  | | 13r  16z | |  | | 11r  16z | |  | |  | |  | |  | | |  |  | | |  | | |  | |  | | 2r  0z | | |  | | |  | | |  | |  | |
| 36 | |  | |  | | 22 | |  | |  | |  | |  | |  | | 48 | |  | |  | |  | |  | | 17a  9d | |  | | |  |  | | |  | | |  | |  | |  | | |  | | |  | | |  | |  | |
| 50 | | 47, 6m | |  | |  | |  | |  | |  | |  | |  | |  | |  | |  | |  | |  | |  | |  | | |  |  | | |  | | |  | |  | |  | | |  | | |  | | |  | |  | |
| 51 | | 65, 6m | |  | |  | |  | |  | |  | |  | |  | |  | |  | |  | |  | |  | |  | |  | | |  |  | | |  | | |  | |  | |  | | |  | | |  | | |  | |  | |
| End-of-life phase | | | | | | | | | | | | | | | | | | | | | | | | | | | | | | | | | | | | | | | | | | | | | | | | | | | | | | | |  |

| Reference | **Seizures (1)** | **Cognitive deficits (2)** | **Drowsiness (3)** | **Dysphagia** | **Headache** | **Confusion (4)** | **Aphasia (5)** | **Motor deficits (6)** | **Fatigue (7)** | **Dyspnea (8)** | **Nausea/ Vomiting (9)** | **Urinary in-continence 10)** | **Pain (11)** | **Aniety/De-pression (12)** | **Anorexia (13)** | **Sensory deficits** | **Dizziness (14)** | **Visual deficits (15)** | **Gait distur-bance** | **Alopecia** | **Skin problems (16)** | **Right left confusion** | **Constipation** | **Diarrhea** | **Dyspepsia** |
| --- | --- | --- | --- | --- | --- | --- | --- | --- | --- | --- | --- | --- | --- | --- | --- | --- | --- | --- | --- | --- | --- | --- | --- | --- | --- |
| 23 | 38m  26w | 38m  48w | 31m  75w | 25w | 40m  35w | 24m  44w | 39m  48w | 42m  41w | 58m  58w | 24w | 19m,n  16w,n | 23m  36w | 10m  12w | 18m,a  18w,a  12m,d  8w,d |  |  |  | 22m  23w |  |  |  |  |  |  |  |
| 37 | 45 | 33 | 87 | 71 | 33 | 29 |  | 51 | 25 | 16 | 20 | 40 | 25 | 9 |  |  | 2 |  |  |  |  |  | 9 |  |  |
| 55 | 65 |  | 95 | 65 | 33 |  |  |  |  |  | 19v |  |  |  |  |  |  |  |  |  | 22 |  |  |  |  |

The symptoms were also described as: (1)‘convulsions’, (2)‘cognitive changes’, ‘cognitive disturbances’, ‘cognitive dysfunction’, ‘progressive cognitive deficits’, ‘cognitive’, ‘mood/cognition changes/ ideomotor slowing’ or ‘cognition’, (3)‘somnolence’, ’drowsiness/progressive loose of consciousness’, ‘loss of consciousness’, ‘altered level of consciousness’ or ‘level of consciousness’, (4)’confusion or agitation’, ‘confusional state’, ’delirium’, or memory loss/confusion’, (5)‘speech deficit’, ‘dysphasia’, ‘language disorder’, ‘communication deficit’, or ‘language deficits’, (6)‘paresis and mobility’, ‘motor paresis’, ‘paresis’, ’ myopathy’, ‘weakness/hemiplegia’, or ‘hemi paresis or paresis’, (7)‘reduced general condition’, ‘fatigue/asthenia’, ‘tiredness’, or ‘asthenia’ (8)‘shortness of breath’, (9)‘gastrointestinal complication’, (10)‘urogenital complications’, or ‘urinary frequency’, (11)‘bodily pain’, (12) ‘psychotic disorders’, (13)‘intake or problems with intake’, ‘lack of appetite’, or ‘appetite’, (14)‘vertigo’ or ‘unsteadiness/ataxia’, (15)’visual’, ‘changes in visual field’, ‘visual disorders’, ‘vision changes’, or ‘visual field disturbances’, (16)‘pruritus/itching’, ‘dermatologic’, or ‘rash’.

a=anxiety, d=depression, ep=early postoperative, fu=during follow up, m=3months before death, n=nausea, pr=presenting, r=radiotherapy/temozolomide, v=vomiting, w=1week before death of patient, z=adjuvant temozolomide, 6m= follow up 6 months postoperative, 12m= follow up 12 months post operative.
